# Apolipoprotein E2/2 (*APOE2/2*) Genotype Is Associated With Reduced Choroidal Thickness

**DOI:** 10.1167/iovs.67.11.38

**Published:** 2026-09-18

**Authors:** Grace A. Borchert, Lino A. F. Ferreira, Shabnam Raji, Jing Yu, Matias Segovia, Robert E. MacLaren, Susan M. Downes, Jasmina Cehajic-Kapetanovic, Kanmin Xue, Samantha R. De Silva

**Affiliations:** 1 Nuffield Laboratory of Ophthalmology, Nuffield Department of Clinical Neurosciences, University of Oxford, Oxford, United Kingdom; 2 Oxford Eye Hospital, Oxford University NHS Foundation Trust, Oxford, United Kingdom; 3 Centre for Human Genetics, Nuffield Department of Medicine, University of Oxford, Oxford, United Kingdom; 4 Department of Statistics, University of Oxford, Oxford, United Kingdom; 5 Great Ormond Street Hospital for Children NHS Foundation Trust, London, United Kingdom

**Keywords:** apolipoprotein E, APOE, age-related macular degeneration, AMD

## Abstract

**Purpose:**

Age-related macular degeneration (AMD) is a leading cause of visual impairment in individuals >50 years. The *APOE2* allele has been associated with increased AMD risk compared to the common *APOE3* allele. However, the retinal phenotype of *APOE2/2* individuals has not been characterized*.* This study aimed to compare the macular structure and function of *APOE2/2* versus *APOE3/3* homozygous individuals.

**Methods:**

Age- and sex-matched participants with *APOE2/2* and *APOE3/3* genotypes were prospectively recruited from the Oxford Biobank. Phenotyping included best-corrected visual acuity (BCVA), low-luminance visual acuity (LLVA), microperimetry, refractive error, multimodal retinal imaging, fasting lipid profile, and EPIC-Norfolk food frequency questionnaires. A linear mixed model of paired eye measurements with a subject-specific random intercept was applied, including age, sex, smoking status and refractive error as covariates. Findings were compared to similar UK Biobank cohorts.

**Results:**

24 participants were recruited: 12 *APOE2/2* and 12 *APOE3/3* (mean age 60.5 ± 5.7 (standard deviation) years, female/male 1:1; 87.5% non-smokers). Serum triglycerides were significantly higher in *APOE2/2* participants (1.8 vs. 1.03 mM, *P* = 0.010). A novel finding of reduced choroidal thickness was found in *APOE2/2* compared to *APOE3/3* participants after multivariate analysis controlling for age, sex, smoking status and refractive error (estimated difference 57.8 µm, *P* = 0.02). Optical coherence tomography segmentation showed a trend toward reduced retinal thickness in *APOE2/2* compared to *APOE3/3* participants, but did not reach statistical significance. No differences in BCVA, LLVA, or retinal sensitivity were detected. Diet and background AMD genetic risk were comparable.

**Conclusions:**

We report a novel genotype-phenotype association of reduced choroidal thickness in individuals homozygous for *APOE2/2* compared with normative *APOE3/3* controls.

Age-related macular degeneration (AMD) is the leading cause of blindness in people over 50 years of age in the developed world.^1^^,^^2^ The hallmark of AMD is drusen which are extracellular, yellow-white lipid and proteinaceous deposits under the retina.^3^ Risk factors for AMD include age, genetic and environmental factors including smoking and dietary lipids.^4^ As a multifactorial disease, AMD pathogenesis appears to involve a complex interplay between aging-related changes in retinal lipid metabolism, oxidative stress, and inflammation.^5^^–^^7^

Variants in complement factor H (*CFH)* and high temperature required factor A1 (*ARMS2/HTRA1*, encoding a serine protease) are both significantly associated with AMD.^8^^,^^9^ The phenotypic features of patients with AMD carrying *CFH* and *ARMS2/HTRA1* variants have been well characterized, whereas other established AMD risk factors such as *APOE2/2* have not been investigated to the same extent.

Apolipoprotein E (APOE) is the primary cholesterol and lipid transporter in the retina and central nervous system. It is the protein component of most lipoproteins, and through binding to the low-density lipoprotein (LDL) receptor facilitates lipid uptake by neuronal cells.^10^^–^^12^ Interestingly, APOE has been shown to be a major constituent of drusen, and structural variants in APOE have been associated with the risk of developing AMD, glaucoma and Alzheimer's disease.^13^^–^^15^ Two single nucleotide polymorphisms (SNPs) in the coding sequence of the *APOE* gene result in amino acid substitutions at positions 112 and 158 in the APOE protein. Combinations of these two SNPs give rise to three allelic variants: *APOE2* (Cys112 Cys158) with an allele frequency of 6.4%, *APOE3* (Cys112 Arg158) with a frequency of 78.3% (i.e., the common allele), and *APOE4* (Arg112 Arg158) with a frequency of 14.5%.^13^^,^^16^ These amino acid substitutions affect the LDL receptor binding property of APOE.^17^ Genome-wide association studies suggest that the *APOE2* allele is associated with increased risk of AMD (odds ratio = 1.12–1.83) but decreased risk of Alzheimer's disease (odds ratio = 0.13 for *APOE2/2* vs. *APOE3/3* homozygotes).^18^^–^^21^ In a Danish cohort study, the *APOE2/*2 genotype was associated with the highest plasma APOE level and had an increased risk of AMD. It was also demonstrated that beyond the established effects of *APOE* polymorphisms, rare functional *APOE* variants also contribute to AMD risk.^15^

The retinal and choroidal phenotype of the *APOE2/2* homozygous genotype has not been well characterized before and may provide further insights into the potential association between *APOE* variants and AMD pathogenesis. In this study, we aimed to characterize the clinical phenotype of *APOE2/2* individuals with reference to age-matched *APOE3/3* controls, including macular structure, function and systemic lipid profile.

## Methods

### Prospective Oxford Biobank Study

A prospective, observational study was performed at the Oxford Eye Hospital, Oxford University Hospitals NHS Foundation Trust, UK, with national research ethics committee approval (REC ref: 23/EE/0207) and in accordance with the Tenets of the Declaration of Helsinki. Participants were identified through the Oxford Biobank and invited to take part between April 2024 and December 2024. The Oxford Biobank is a prospective community cohort of >8000 individuals who have had a baseline screening visit, donated DNA for genetic testing and given informed consent to be re-approached for research studies (REC ref: 18/SC/0588).^22^

For this study, inclusion criteria were *APOE2/2* or *APOE3/3* homozygous genotype, whereas individuals with ocular diseases that might interfere with clinical assessment or retinal phenotyping were excluded. During their visit, participants underwent early-morning fasting blood sampling for evaluation of lipid profile. A clinical eye assessment consisted of functional testing including best-corrected visual acuity (BCVA), low-luminance visual acuity (LLVA) and intraocular pressure (IOP) measurement using an iCare tonometer. In addition, structural assessment included multimodal retinal imaging with Optomap ultra-widefield scanning laser ophthalmoscopy (Optos Inc, Dunfermline, UK), blue-light autofluorescence (BAF) and spectral-domain optical coherence tomography (OCT) and OCT angiography (Heidelberg Spectralis HRA; Heidelberg Engineering GmbH, Heidelberg, Germany). Mesopic microperimetry (Macular Integrity Assessment; Centervue SpA, Padua, Italy) was performed using a 4-2 strategy for each eye and then compared to a normal reference database and to the age- and sex-matched *APOE3/3* participants. Each participant also completed a validated food frequency questionnaire (EPIC-Norfolk FFQ).^23^ The questionnaire was encoded from 1 to 9 based on frequency (part 1) and food category (part 2). The encoded response was input into the FFQ EPIC Tool for Analysis (FETA) software for micronutrient quantification output before then being decoded.

A custom-built analysis tool was used to automate retinal thickness measurements from multiple OCT slices in the Heidelberg Eye Explorer software and these were mapped using the Early Treatment Diabetic Retinopathy Study (ETDRS) grid tool.^24^ The ETDRS grid was separated into three concentric rings for analysis: central 1 mm, inner 3 mm, and outer 6 mm rings. The measurement for each ring was the mean of the four quadrants: superior, temporal, inferior, and nasal.

Choroidal thickness was measured at the sub-foveal point and at one B-scan immediately superior and inferior to the fovea. These three measurements were to account for anatomical choroidal variation. To assess robustness, choroidal thickness measurements were repeated by a second independent and blinded grader. The linear mixed model used for analysis included choroidal measurements that were averaged across the graders.

LLVA was measured monocularly following refraction, using a 2.0 log unit neutral density filter placed in front of the participants eye. Room lights were switched off and acuity was measured using the retro-illuminated standard ETDRS visual acuity chart (luminance 160 cd/m^2^). The Low luminance deficit was calculated by determining the difference between BCVA and LLVA.

Microperimetry was performed using the Macular Integrity Assessment after 20 minutes of formal dark adaptation and pharmacological pupil dilation using Tropicamide 0.5% and Phenylephrine 2.5%. For mesopic microperimetry, a standard 10-2 test grid and 4-2 bracketing threshold strategy was chosen, and examination involved presentation of Goldmann size III stimuli of various intensities (0–318 cd/m^2^), presented for 200 ms, on a mesopic background (1.27 cd/m^2^). The overall dynamic testing range was 0–36 dB. Microperimetry reliability was assessed based on the bivariate contour ellipse area (BCEA). Participants were considered unreliable if BCEA95 >4° or they had a condition that interfered with microperimetry.^2^^,^^25^

### Analysis of *APOE* Homozygous Individuals in the UK Biobank

The UK Biobank is a community-based prospective cohort study in the UK with around 500,000 participants. A subset of participants in the UK Biobank study had ophthalmic assessments including BCVA, IOP and OCT (3D OCT-1000; Topcon Healthcare, Oakland, NJ, USA). All participants were genotyped for around 800,000 genetic variants with the UK Biobank Axiom Array or UK BiLEVE Axiom Array from Affymetrix as previously described.^26^

Data were accessed through the UK Biobank Research Analysis Platform. Statistical analysis and visualization of data were performed with the R programming language. Genotyping data were obtained for all participants based on *APOE2/2* and *APOE3/3* homozygous genotype. Participants were excluded if they (i) were aged less than 50 years, (ii) had ocular co-morbidities including diabetic retinopathy, glaucoma or eye trauma (as per information available within the UK Biobank), (iii) did not have OCT imaging performed, or (iv) had inadequate OCT imaging quality (defined as image quality <45 based on the threshold published by Topcon, and ILM (Inner limiting membrane) indicator <0, measuring the minimum localized edge strength at the ILM boundary, or validity count <744, which represents the extent of clipping in the OCT scan's *z*-dimension (threshold is the bottom 10% of quality)). These strict imaging-quality criteria were to ensure that all participants had adequate OCT segmentation to enable comparisons between genotypes and only these eyes were included for analysis. The UK Biobank provides numerical data based on automated OCT segmentation by the Topcon Advanced Boundary Segmentation software. However, no choroidal thickness data were available.

### Statistical Analysis

To assess the effect of *APOE* genotype on choroidal and retinal thickness in the Oxford Biobank, we began by comparing the average retinal thickness in each ETDRS segment across genotype groups using simple *t*-tests (Supplementary Fig. S3; paired eye measurements were averaged for each subject). In our main analysis, we focused on retinal thickness in the inner 3 mm and outer 6 mm ETDRS rings and choroidal thickness. After performing simple *t*-tests on averaged measurements, we applied a linear mixed model of paired thickness with genotype and covariates as fixed effects and a subject-specific random intercept to account for the within-subject correlations caused by the paired design, as recommended in the literature (*P*-values were computed based on parametric bootstrapping with 10,000 replicates).^27^^,^^28^ As a robustness check, we also verified that the simpler approach of averaging eye measurements for each subject and using standard linear regression yielded comparable results but opted for the mixed model because it made better use of all the information in the data.

We included the following as covariates in these models: age, sex, smoking status, and refractive error (spherical equivalent, available for each eye and processed as a paired variable). Including additional measurements from the lipid blood and FFQ profile (cholesterol, total fat, monosaturated fats, polysaturated fats and saturated fats) did not yield meaningfully different conclusions. For our analysis of the UK Biobank data, we averaged the measurements between eyes per subject and used simple *t*-tests to analyze nine retinal thickness measures (Supplementary Fig. S7) followed by linear regression controlling for demographic variables.

Data were plotted with GraphPad Prism 9 (GraphPad software, San Diego, CA, USA). Statistical analyses were performed using R version 4.1.2, with mixed models fitted with the package lme4 and parametric bootstrapping performed with pbkrtest.^27^^,^^28^

## Results

There were 44 participants who were identified to have the *APOE2/2* genotype in the Oxford Biobank. Of these, 12 agreed to participate in the study and thus underwent ophthalmic examination, retinal assessment, fasting lipid profile, and dietary questionnaire. Twelve control *APOE3/3* participants who were age- and sex-matched were also recruited (Supplementary Fig. S1).

The mean age of all participants was 60.5 years and there were 50% females and 50% males in the recruited cohort (Table, Supplementary Table S1). Among the *APOE2/2* participants, 91.7% were non-smokers, and among the *APOE3/3* participants, 83.33% were non-smokers. BCVA, LLVA, and IOP were comparable between the *APOE2/2* and *APOE3/3* groups*.* There appeared to be a stronger family history of AMD among the *APOE2/2* participants (30% or three out of 10 participants; two had unknown family history because of adoption) relative to *APOE3/3* participants (8.33%). No participants were pseudophakic at the time of recruitment. The mean refractive error for *APOE2/*2 was −1.7 D (standard deviation [SD] = 2.2 D) and for *APOE3/*3 was 0.08 D (SD = 1.1 D) (*P* = 0.04).

**Table.** Baseline Demographics of *APOE2/2* and *APOE3/3* Participants

	*APOE2/2*	*APOE3/3*	*P* Value
Mean age at recruitment, years (SD)	60.5 (5.12)	60.5 (5.14)	0.9201
Sex			0.9999
Female	6 (50%)	6 (50%)	
Male	6 (50%)	6 (50%)	
Never smoked	11 (91.7%)	10 (83.33%)	0.2179
Previous smoker	0	2 (16.67%)	NA
Current smoker	1 (8.3%)	0	NA
Mean BCVA, logMAR (SD)	89.88 (4.05)	88.12 (7.19)	0.5965
Mean LLVA, logMAR (SD)	77.46 (3.82)	75.73 (9.42)	0.8219
Mean IOP, mm Hg (SD)	15.58 (3.99)	15 (4.25)	0.5922

Other AMD genetic risk factors, including *CFH, C5, ARMS2, C3, HTRA1* and *LIPC*, were comparable between *APOE2/2* and *APOE3/3* participants (Supplementary Fig. S2). Food frequency questionnaire responses were encoded according to standardized categories, analyzed using FETA software and converted into micronutrients (such as total fat, monounsaturated fatty acids, polysaturated fatty acids, saturated fatty acids) (Supplementary Table S2). Comparison of dietary intake between groups showed no significant difference.

**Figure 1.**
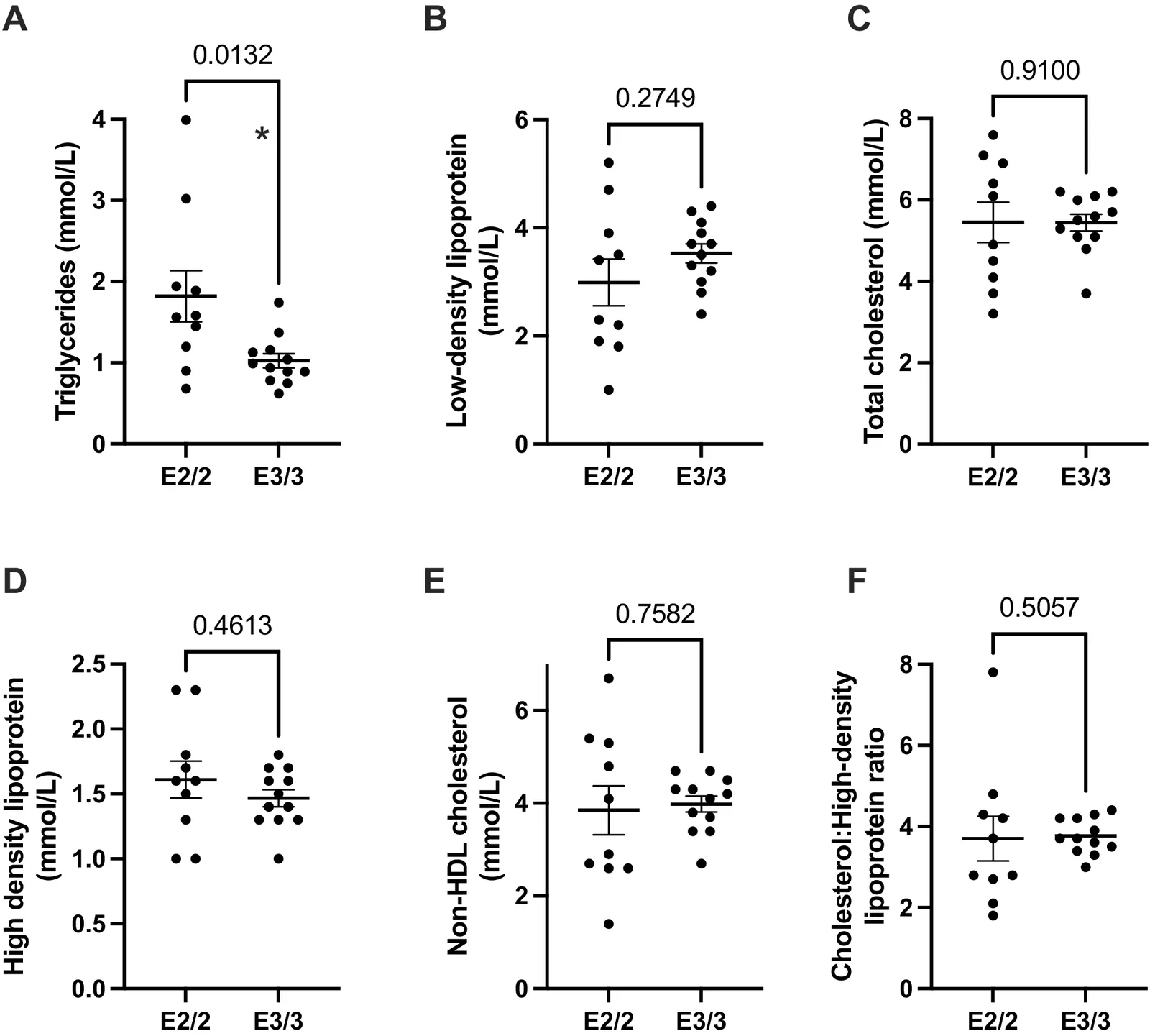
Lipid profile comparing *APOE2/2* to *APOE3/3* participants. **(A)** Triglycerides **(B)** Low-density lipoprotein (LDL), **(C)** Total cholesterol, **(D)** High density lipoprotein, **(E)** Non-HDL cholesterol, and **(F)** Cholesterol:High-density lipoprotein (HDL) ratio. This excluded two participants who were prescribed atorvastatin (two in the *APOE2/2* cohort) to avoid confounding the lipid profile. A *t*-test was used and a difference was considered significant when *P*-value < 0.05 (represented by *).

Considering the functional role of apolipoprotein E and the effect of *APOE2/2* homozygosity on its lipid binding and uptake characteristics, fasting lipid profile was compared against *APOE3*/*3* (Fig. 1). It was demonstrated that systemic triglyceride levels were significantly higher among *APOE2/2* (1.8 mM) participants compared to *APOE3/3* (1.0 mM) participants (*P* = 0.013). There were no significant differences in total cholesterol, LDL, high-density lipoprotein (HDL), non-HDL cholesterol, and cholesterol: HDL ratio.

**Figure 2.**
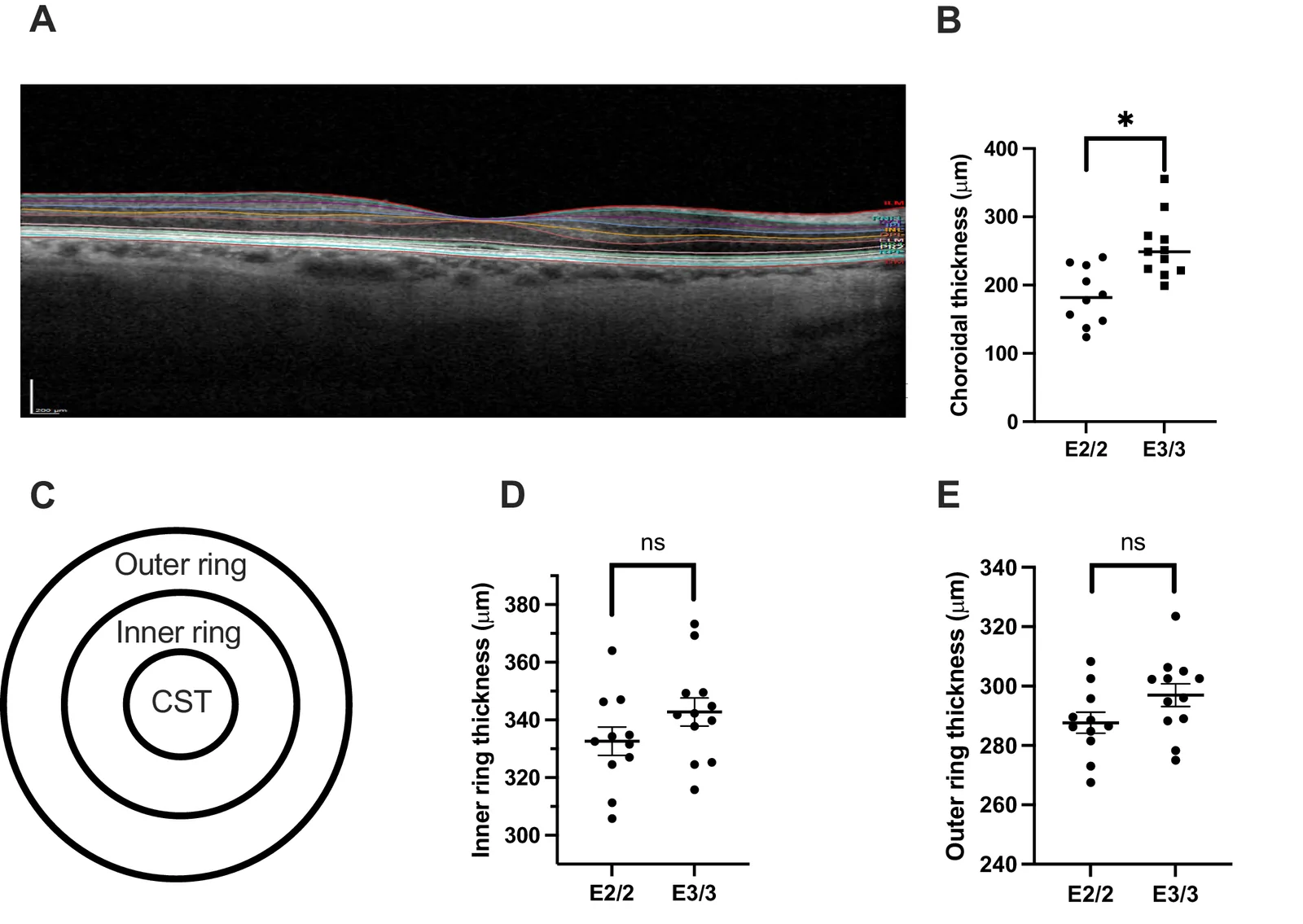
Sub-foveal choroidal and retinal thinning in *APOE2/2* participants compared to *APOE3/3*. **(A)** An example OCT from an *APOE2/2* participant illustrates automated segmentation of retinal layers, **(B)** choroidal thickness averaged from three points (subfoveal, and at the subfoveal location in OCT slices immediately superior and inferior to the fovea) demonstrated thinning in *APOE2/2* participants compared to *APOE3/3* participants, **(C)** illustration of the central subfield thickness (CST), inner 3 mm and outer 6 mm ETDRS rings, comparison of **(D)** inner ring thickness, and **(E)** outer ring thickness in *APOE2/2* and *APOE3/3* participants. A difference was considered significant when *P*-value < 0.05 (represented by *).

Central choroidal thickness was found to be significantly reduced in *APOE2/2* participants (183 µm, SD = 42) compared to *APOE3/3* participants (253 µm, SD = 48) (Fig. 2); after multivariate analysis controlling for age, sex, smoking status and refractive error, the estimated difference was 57.82 µm (*P* = 0.02). To assess robustness, choroidal thickness measurements were repeated by a second independent and blinded grader, yielding highly correlated results (Supplementary Fig. S4). A linear mixed model with choroidal measurements averaged across graders resulted in a similar estimated effect of 57.65 µm (*P* = 0.02). OCT segmentation analysis showed a mildly reduced retinal thickness in the inner 3 mm ETDRS ring in *APOE2/2* compared to *APOE3/3* participants (mean 332 µm versus 345 µm, respectively), as well as the outer 6 mm ETDRS ring (mean 287 µm vs. 298 µm, respectively) (Fig. 2). However, these did not reach statistical significance either in the simple *t*-test for difference in means or after multivariate analysis (estimated difference = 10.48 µm, *P* = 0.09 for the inner ring; estimated difference = 9.62 µm, *P* = 0.09 for the outer ring).

**Figure 3.**
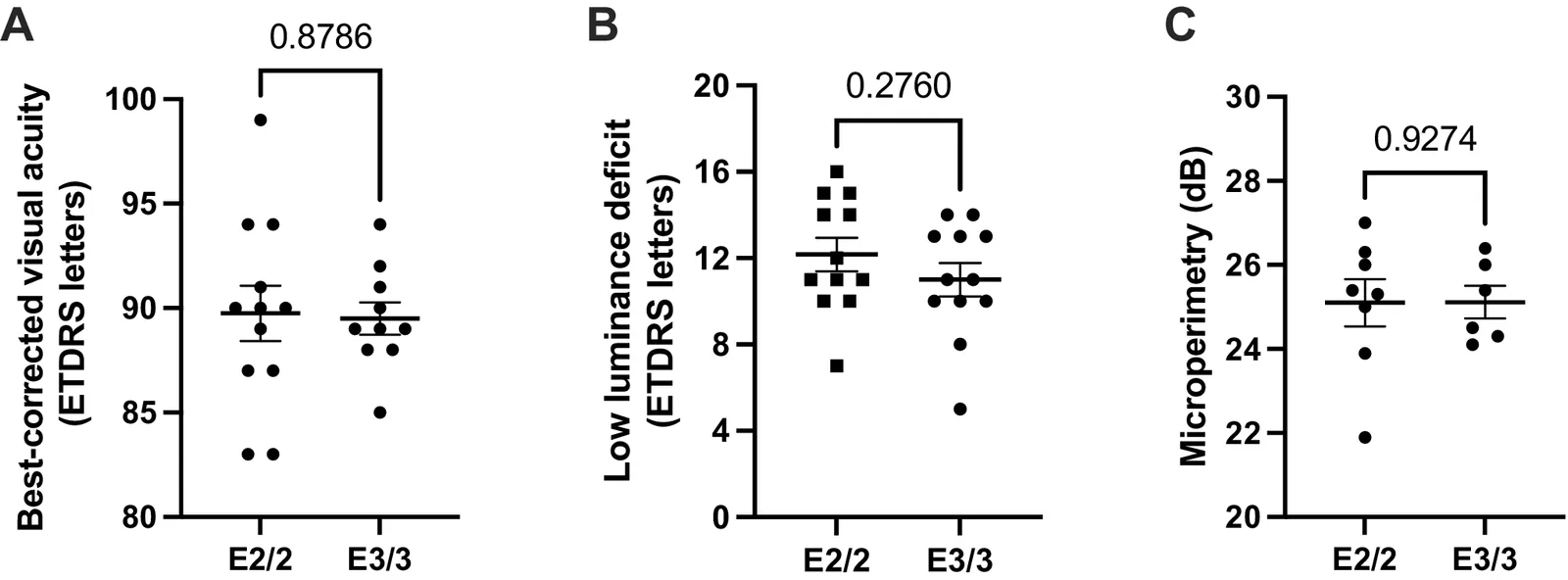
Functional measures. Comparison of **(A)** best corrected visual acuity (BCVA), **(B)** low luminance deficit (LLD), defined as the difference between BCVA and LLVA, and **(C)** microperimetry in *APOE2/2* and *APOE3/3* participants. A *t*-test was used and a difference was considered significant when *P*-value < 0.05 (represented by *).

We assessed if the structural changes correlated with any changes in visual function as measured by microperimetry. No significant functional differences were detected (Fig. 3, Supplementary Figs. S5 and S6).

**Figure 4.**
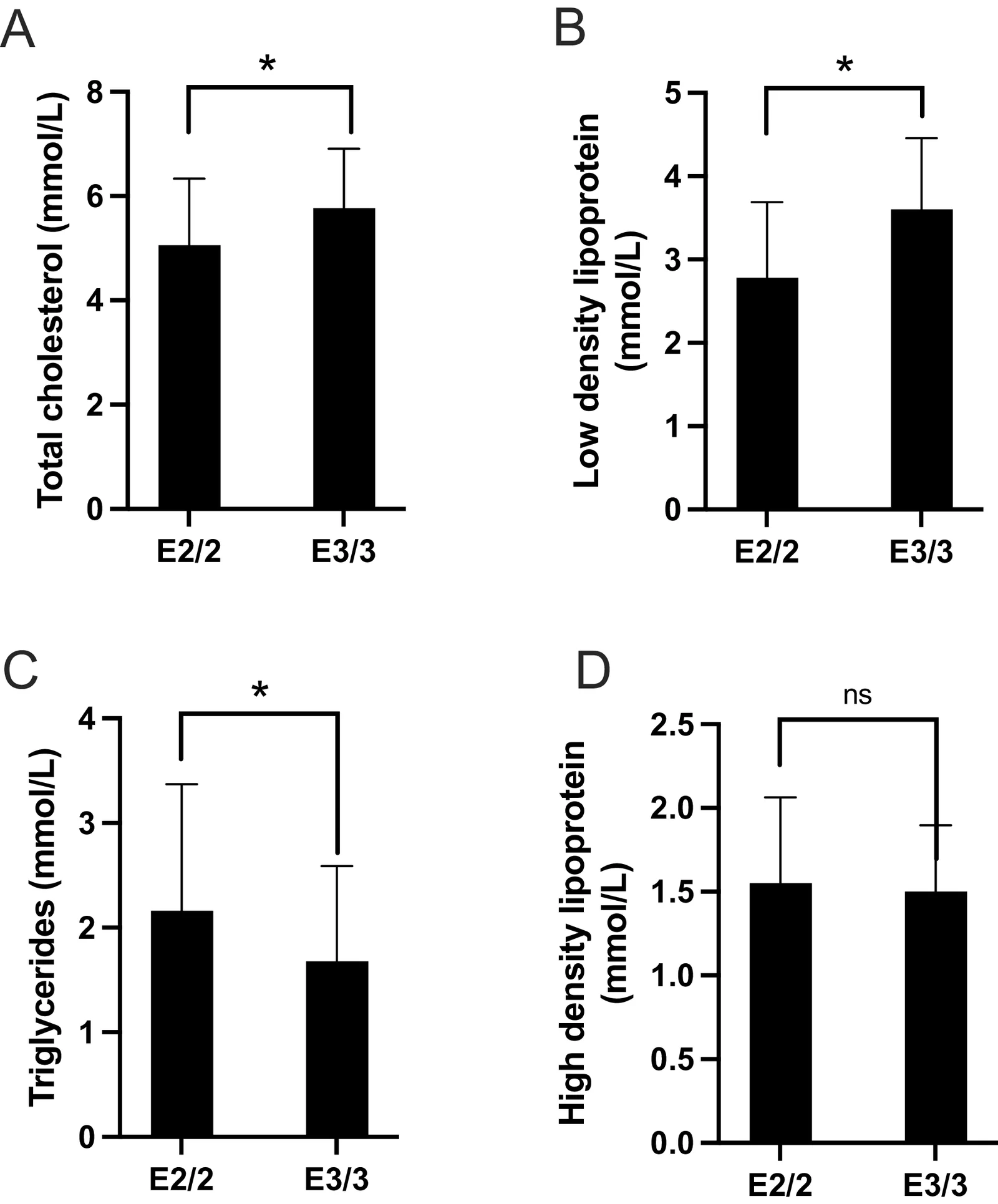
Comparison of lipid profile between UK Biobank participants with APOE2/2 and APOE3/3 genotypes. **(A)** Total Cholesterol levels. **(B)** Low-density lipoprotein (LDL). **(C)** Triglyceride levels **(D)** High-density lipoprotein (HDL). The *P*-value was considered significant when <0.05 and is represented by * annotation.

Similarly, these outcome measures were also investigated in the UK Biobank dataset. There were 245 *APOE2/2* participants and 21,750 *APOE3/*3 participants with a mean age at recruitment of 60.4 years (SD = 5.14) and 45% females. There was no significant difference in BCVA between *APOE2/2* and *APOE3/3* participants. However, LLVA and microperimetry were not performed as part of UK Biobank assessments. *APOE2/2* participants had significantly lower total cholesterol (5.06 vs. 5.77 mM, *P* < 2e–16), lower LDL (2.78 vs. 3.6 mM, *P* < e−16) and higher triglycerides (2.16 vs. 1.68 mM, *P* < 8.7e–10) than *APOE3/3* participants (Fig. 4). Meanwhile, no significant difference was found in HDL, which was consistent with the Oxford Biobank participants. The computed total retinal thickness was comparable between *APOE2/2* and *APOE3/3* participants in the UK Biobank (Supplementary Fig. S7; linear regression analyses also did not detect significant differences). Choroidal thickness was not measured as part of the UK Biobank dataset.

## Discussion

The retinal and choroidal phenotype of *APOE2/2* homozygous individuals has not been reported before. Although individuals with genetic risk alleles in the complement pathway have been well characterized clinically, AMD genetic risk factors involving lipid metabolism have not been extensively explored, and there are no clinical studies investigating retinal or choroidal thickness. This is the first study to characterize in detail the choroidal and retinal phenotype of *APOE2/2* compared to age- and sex-matched *APOE3/3* controls. We demonstrated choroidal thinning associated with the *APOE2/2* genotype compared to age- and sex-matched *APOE3/3* individuals after correcting for a range of relevant variables (Fig. 2). These data are also novel because choroidal thickness was not assessed as part of the UK Biobank, whilst enhanced-depth OCT enabled us to quantify it among the Oxford Biobank participants. In addition, a mild reduction in retinal thickness was detected in *APOE2/2* participants but after adjusting for refractive error, age, sex, and smoking this did not reach statistical significance, which was consistent with analysis of data available from the UK Biobank. In addition, *APOE2/2* participants (30%) were more likely to have a family history of AMD (30%) compared to *APOE3/3* participants (8.33%). One of the *APOE2/2* participants was informed of a new diagnosis of early AMD with multiple small drusen.

APOE plays a central role in lipid transport, inflammation, and extracellular remodelling.^4^^,^^13^ In the outer retina, photoreceptor outer segments are shed daily in a highly regulated process and subsequently phagocytosed by the retinal pigment epithelium (RPE), with APOE contributing to the recycling of lipid components. The single amino acid substitution defining the APOE2 isoform (Arg158Cys) alters protein structure and impairs LDL receptor binding relative to the E3 and E4 isoforms, resulting in reduced clearance of triglyceride-rich lipoprotein remnant particles.^10^ Consequently, *APOE2/2* individuals exhibit elevated triglyceride levels (1.821 mM) compared to *APOE3/3* individuals (1.025 mM), a finding observed in both the Oxford Biobank (*P* = 0.0132) and UK Biobank (*P* = 8.7 e – 10). Consistent with this, *APOE2/2* status has previously been associated with elevated triglycerides and reduced LDL levels in both humans and mice, while *apoe* knockout mice fed a high-fat diet demonstrate increased oxidative stress and inflammation—pathological features reminiscent of AMD.^29^ In addition, a large Danish cohort study of >100,000 individuals showed that *APOE2/2* is associated with higher plasma APOE level, which appears to be associated with greater risk for AMD.^15^

Our study is the first to report choroidal thinning in association with the *APOE2/*2 genotype. AMD is associated with thinning of the subfoveal choroid, which may reflect vascular insufficiency and a relative deficiency in oxygen and nutrient delivery to the RPE and photoreceptors.^30^^,^^31^ Supporting this hypothesis, primary human RPE cultures exposed to hypoxia show increased apical versus basal secretion of APOE, a mechanism that may contribute to subretinal drusenoid deposit formation and accelerated AMD progression.^32^ Together, dysregulation of RPE metabolism and impaired retinal lipid clearance associated with *APOE2/2* may promote chronic local inflammation—a hallmark of AMD—and contribute to secondary retinal and choroidal thinning.^4^ Choroidal thinning has been linked to age and myopia; however, in this study, participants were age-matched, and adjustment for refractive error still revealed a significantly reduced central choroidal thickness in *APOE2/2* participants.^33^

Meanwhile, our study detected a mild but non-significant reduction in retinal thickness among the *APOE2/2* participants compared with *APOE3/3* controls. OCT-derived metrics have previously been used to characterize macular thickness changes associated with AMD risk factors. Mutations in the *CFI* gene encoding complement factor I, a negative regulatory enzyme of complement, has been associated with reduced macular thickness and AMD in the UK Biobank at a mean age of 55 years.^34^ Carriers of rare variants in the *CFH* gene had a thinner retina based on total macular volume and inner retinal layer volume.^35^ However, this was in a cohort with a mean age of 73 years. A genome-wide association analysis identified 139 loci associated with reduced macular thickness in the UK Biobank. However, their analysis of *APOE* was not based on the combined rs7412 and rs429358 haplotype for *APOE* alleles, and only individual SNPs were assessed.^36^ The rs429358 haplotype was individually considered for association with macular thickness but did not demonstrate a significant difference.^37^

There are a number of limitations to our study. In particular, there was a limited number of participants with the genotype of interest (*APOE2/2*) able to participate in the study from the Oxford Biobank. The recruited cohort had an average age of 60.5 years, which is relatively young for the assessment of early AMD features, although this would make the choroidal thinning even more notable. It would be valuable to investigate longitudinal follow up (e.g., over 10 years) to see if the choroidal and macular thickness differences become more pronounced over time. Moreover, given that an early biomarker of AMD is impaired dark adaptation, this would be a valuable functional assessment to incorporate into future assessments.^38^ Finally, the current UK Biobank data were limited to derived automated Topcon segmentation values without direct visualization of OCT images and no choroidal thickness data were available*.* This may improve in the future with ongoing re-evaluation of the UK Biobank cohort with improved retinal imaging.

## Conclusions

We report a novel genotype-phenotype association between the *APOE2/2* genotype and reduced choroidal thickness, which may represent an early biomarker of AMD risk. Longitudinal research is needed to further characterize the association between *APOE* alleles and AMD pathogenesis. We also demonstrated increased serum triglycerides among *APOE2/2* participants in the Oxford Biobank, as well as decreased LDL and total cholesterol in corresponding UK Biobank participants. These changes in lipid profile may affect retinal lipid metabolism and formation of AMD-associated deposits, including distinct drusen subtypes that exhibit differing associations with choroidal thickness. Understanding these systemic and local lipid processing pathways could improve our understanding of AMD pathogenesis and enable novel treatment strategies.

## Supplementary Material

Supplement 1
