# Supplementary material for: Apolipoprotein E2/2 (APOE2/2) Genotype Is Associated With Reduced Choroidal Thickness
Source: Invest Ophthalmol Vis Sci. 2026 Sep 18;67(11):38. doi: 10.1167/iovs.67.11.38 (PMC13614145; doi:10.1167/iovs.67.11.38)
Supplement: Supplementary file 1 [file iovs-67-11-38_s001.docx]

**Supplementary** **Figure 1. Flowchart of participant recruitment from the Oxford Biobank**.

**Supplementary Figure 2. Genetic risk factors for age-related macular degeneration background.** The shade of each cell represents the average genotype risk allele presence between the *APOE2/2* and *APOE3/3* genotype cohorts. Wild type is 0 and is blue, one risk allele is 0.5 and represented by white, and two risk alleles is 1 and represented by red.

**Supplementary Table 1. Demographics of recruited participants**. *APOE2/2* are participants 1 to 12 and *APOE3/3* participants are 13 to 24. BCVA = Best corrected visual acuity. LLVA = low luminance visual acuity. IOP = intraocular pressure. OS = left eye. OD = right eye.

| **Patient** | **Age (years)** | **Sex** | **BCVA OD**  **(letters)** | **BCVA OS**  **(letters)** | **LLVA OD**  **(letters)** | **LLVA OS**  **(letters)** | **IOP OD**  **(mmHg)** | **IOP OS**  **(mmHg)** |
| --- | --- | --- | --- | --- | --- | --- | --- | --- |
| 1 | 61 | Female | 94 | 84 | 82 | 74 | 10 | 10 |
| 2 | 56 | Male | 90 | 89 | 79 | 74 | 17 | 18 |
| 3 | 62 | Female | 87 | 87 | 71 | 80 | 25 | 25 |
| 4 | 56 | Male | 99 | 98 | 85 | 82 | 19 | 20 |
| 5 | 49 | Female | 90 | 90 | 80 | 77 | 15 | 17 |
| 6 | 63 | Male | 89 | 93 | 75 | 77 | 17 | 18 |
| 7 | 59 | Male | 94 | 94 | 79 | 80 | 12 | 10 |
| 8 | 55 | Male | 91 | 91 | 80 | 83 | 14 | 15 |
| 9 | 67 | Male | 87 | 88 | 76 | 71 | 14 | 12 |
| 10 | 67 | Female | 90 | 88 | 78 | 78 | 12 | 10 |
| 11 | 68 | Female | 83 | 89 | 72 | 77 | 11 | 12 |
| 12 | 63 | Female | 85 | 85 | 77 | 75 | 10 | 13 |
| 13 | 62 | Female | 89 | 88 | 84 | 74 | 17 | 17 |
| 14 | 57 | Male | 88 | 85 | 80 | 76 | 10 | 13 |
| 15 | 63 | Female | 89 | 88 | 76 | 80 | 11 | 13 |
| 16 | 50 | Female | 93 | 89 | 79 | 79 | 17 | 17 |
| 17 | 57 | Male | 90 | 94 | 80 | 80 | 15 | 15 |
| 18 | 59 | Male | 91 | 92 | 77 | 79 | 15 | 15 |
| 19 | 64 | Male | 92 | 91 | 79 | 79 | 15 | 15 |
| 20 | 54 | Male | 95 | 92 | 82 | 83 | 15 | 14 |
| 21 | 63 | Female | 86 | 60 | 76 | 38 | 14 | 9 |
| 22 | 68 | Female | 85 | 87 | 75 | 75 | 13 | 14 |
| 23 | 65 | Male | 76 | 88 | 65 | 70 | 16 | 16 |
| 24 | 90 | Female | 90 | 92 | 81 | 82 | 24 | 28 |

**Supplementary table 2. Micronutrients intake calculated from analysis of dietary questionnaire in *APOE2/2* compared to *APOE3/3* participants*.***

|  | ***APOE2/2* average** | ***APOE2/2* SD** | ***APOE3/3* average** | ***APOE3/3* SD** |
| --- | --- | --- | --- | --- |
| **Alpha carotene** | 433 | 204 | 718 | 810 |
| **Alcohol** | 6 | 7 | 6 | 7 |
| **Beta carotene** | 3293 | 1161 | 4209 | 2601 |
| **Calcium** | 727 | 312 | 574 | 153 |
| **Carotene - total** | 3732 | 1317 | 4748 | 2984 |
| **Carbohydrate - total** | 235 | 133 | 173 | 49 |
| **Cholesterol** | 327 | 227 | 270 | 117 |
| **Chloride** | 4655 | 2141 | 3360 | 815 |
| **Copper** | 1 | 1 | 1 | 0 |
| **Englyst Fibre** | 18 | 6 | 17 | 4 |
| **Iron** | 12 | 5 | 10 | 2 |
| **Total folate** | 280 | 69 | 250 | 42 |
| **Carbohydrate - fructose** | 25 | 10 | 20 | 10 |
| **Carbohydrate - galactose** | 0 | 1 | 1 | 1 |
| **Carbohydrate - glucose** | 24 | 10 | 18 | 7 |
| **Iodine** | 128 | 67 | 110 | 37 |
| **Potassium** | 3497 | 1179 | 3204 | 654 |
| **Energy_kcal** | 2106 | 1265 | 1603 | 357 |
| **Energy_kj** | 8833 | 5290 | 6729 | 1499 |
| **Carbohydrate - lactose** | 6 | 5 | 4 | 2 |
| **Carbohydrate - maltose** | 4 | 4 | 2 | 1 |
| **Magnesium** | 330 | 122 | 295 | 58 |
| **Manganese** | 4 | 2 | 3 | 1 |
| **Sodium** | 3152 | 1532 | 2323 | 554 |
| **Niacin** | 23 | 10 | 23 | 5 |
| **Phosphorus** | 1295 | 581 | 1145 | 229 |
| **Protein** | 80 | 38 | 73 | 17 |
| **Vitamin A - retinol** | 642 | 814 | 615 | 548 |
| **Vitamin A - retinol equivalents** | 1275 | 817 | 1405 | 660 |
| **Vitamin B2 - riboflavin** | 2 | 1 | 1 | 0 |
| **Selenium** | 64 | 23 | 57 | 18 |
| **Carbohydrate - starch** | 111 | 62 | 86 | 24 |
| **Carbohydrate - sucrose** | 55 | 49 | 36 | 15 |
| **Vitamin B1 - thiamine** | 1 | 0 | 1 | 0 |
| **Nitrogen** | 13 | 6 | 12 | 3 |
| **Carbohydrate - sugars** | 118 | 70 | 82 | 29 |
| **Vitamin B12 - cobalamin** | 7 | 4 | 6 | 4 |
| **Vitamin B6 - pyridoxine** | 2 | 1 | 2 | 0 |
| **Vitamin C - ascorbic acid** | 120 | 41 | 104 | 42 |
| **Vitamin D** | 4 | 2 | 3 | 2 |
| **Vitamin E** | 16 | 9 | 12 | 2 |
| **Zinc** | 9 | 5 | 8 | 2 |
| **Fat - total** | 96 | 72 | 69 | 18 |
| **MUFA - total** | 37 | 28 | 27 | 7 |
| **PUFA - total** | 18 | 10 | 12 | 4 |
| **Saturated fatty acids (SFA)** | 34 | 30 | 24 | 7 |
| **Alcoholic beverages** | 91 | 108 | 109 | 124 |
| **Cereals and cereal products** | 247 | 178 | 220 | 102 |
| **Eggs and egg dishes** | 30 | 21 | 20 | 18 |
| **Fats and oils** | 23 | 24 | 15 | 9 |
| **Fish & fish products** | 55 | 41 | 50 | 28 |
| **Fruit** | 255 | 168 | 228 | 128 |
| **Meat and meat products** | 90 | 92 | 106 | 61 |
| **Milk and milk products** | 90 | 53 | 94 | 54 |
| **Non-alcoholic beverages** | 891 | 491 | 851 | 495 |
| **Nuts and seeds** | 22 | 28 | 17 | 14 |
| **Potatoes** | 71 | 34 | 58 | 35 |
| **Soups & sauces** | 134 | 151 | 59 | 29 |
| **Sugars; preserves and snacks** | 67 | 92 | 30 | 27 |
| **Vegetables** | 298 | 105 | 299 | 90 |

**Supplementary Figure 3.** Comparison of retinal thickness quadrants of the inner 3mm ETDRS ring and outer 6mm ETDRS ring on OCT scans according to genotype. (A) Inner inferior thickness, (B) Inner nasal thickness, (C) Inner temporal thickness, (D) Inner superior thickness, (E) Outer inferior thickness, (F) Outer nasal thickness, (G) Outer temporal thickness, and (H) Outer superior thickness. A linear mixed model of paired thickness with genotype and covariates as fixed effects was performed. A difference was considered significant when p-value<0.05 (represented by *).

**Supplementary Figure 4.** Choroidal thickness measurements by two independent blinded graders. The Intergrader agreement was excellent (intraclass correlation coefficient was 0.96) and standard error of measurement was 11.75$\mu m$ indicating low measurement error between graders.

**Supplementary Figure 5.** Flow chart illustrating inclusion and exclusion of participants who had microperimetry testing.

**Supplementary Figure 6.** Reliability of microperimetry based on genotype and operator. (A) BCEA95 in *APOE2/*2 and *APOE3/3* participants*.* BCEA95 is 95% bivariate contour ellipse area which is a statistical metric of the eye fixation. It is the size of an ellipse that contains 95% of the eye’s fixation points during microperimetry. (B) Interoperator comparison of BCEA95.

**Supplementary Figure 7.** Comparison of retinal thickness in the central 1mm subfield, inner 3mm and outer 6mm ETDRS rings on OCT scans according to *APOE* genotype in UK Biobank participants. (A) Central subfield thickness, (B) Inner inferior thickness, (C) Inner nasal thickness, (D) Inner temporal thickness, (E) Inner superior thickness, (F) Outer inferior thickness, (G) Outer nasal thickness, (H) Outer temporal thickness, and (I) Outer superior thickness.
